# Supplementary material for: DeepExtremeCubes: Earth system spatio-temporal data for assessing compound heatwave and drought impacts
Source: Sci Data. 2025 Jan 25;12:149. doi: 10.1038/s41597-025-04447-5 (PMC11763250; doi:10.1038/s41597-025-04447-5)
Supplement: Supplementary file 1 — Supplementary Information [file 41597_2025_4447_MOESM1_ESM.pdf]

Supplementary Information

Table of contents

Table s1. The information of selected land covers in ESA CCI map.......................................1

Figure s1.Three land cover maps extracted from the CCI map..2

Figure s2. Event days map...3

Land cover	CCI ID	Category and description
Broad-leaved trees	50	Tree cover, broad-leaved, evergreen, closed to open (>15%);0;100;0
	60	Tree cover, broad-leaved, deciduous, closed to open (>15%);0;160;0
	61	Tree cover, broad-leaved, deciduous, closed (>40%);0;160;0
	62	Tree cover, broad-leaved, deciduous, open (15-40%);170;200;0
Needle-leaved trees	70	Tree cover, needle-leaved, evergreen, closed to open (>15%);0;60;0
	71	Tree cover, needle-leaved, evergreen, closed (>40%);0;60;0
	72	Tree cover, needle-leaved, evergreen, open (15-40%);0;80;0
	80	Tree cover, needle-leaved, deciduous, closed to open (>15%);40;80;0
	81	Tree cover, needle-leaved, deciduous, closed (>40%);40;80;0
	82	Tree cover, needle-leaved, deciduous, open (15-40%);40;100;0
Mixed trees	90	Tree cover, mixed leaf type (broadleaved and needle-leaved)
Grassland	130	Grassland
Bare area	200	Bare area
Urban area	190	Urban area

Table s1. The information of selected land covers in ESA CCI map.

Considering the substantial size of the land cover map with a 300-meter spatial resolution and the Arctic and Antarctic regions not being our research area of interest, we confined our analysis to the region between 85° N and 55° S, encompassing the targeted vegetation. This reduction in scope decreased the data volume from 62.57 GiB to 48.67 GiB. Furthermore, when the computation of two mixed land cover maps exceeded the available RAM capacity, we divided the global map into ten segments based on their longitudinal spans. This segmentation facilitated the handling of large datasets.

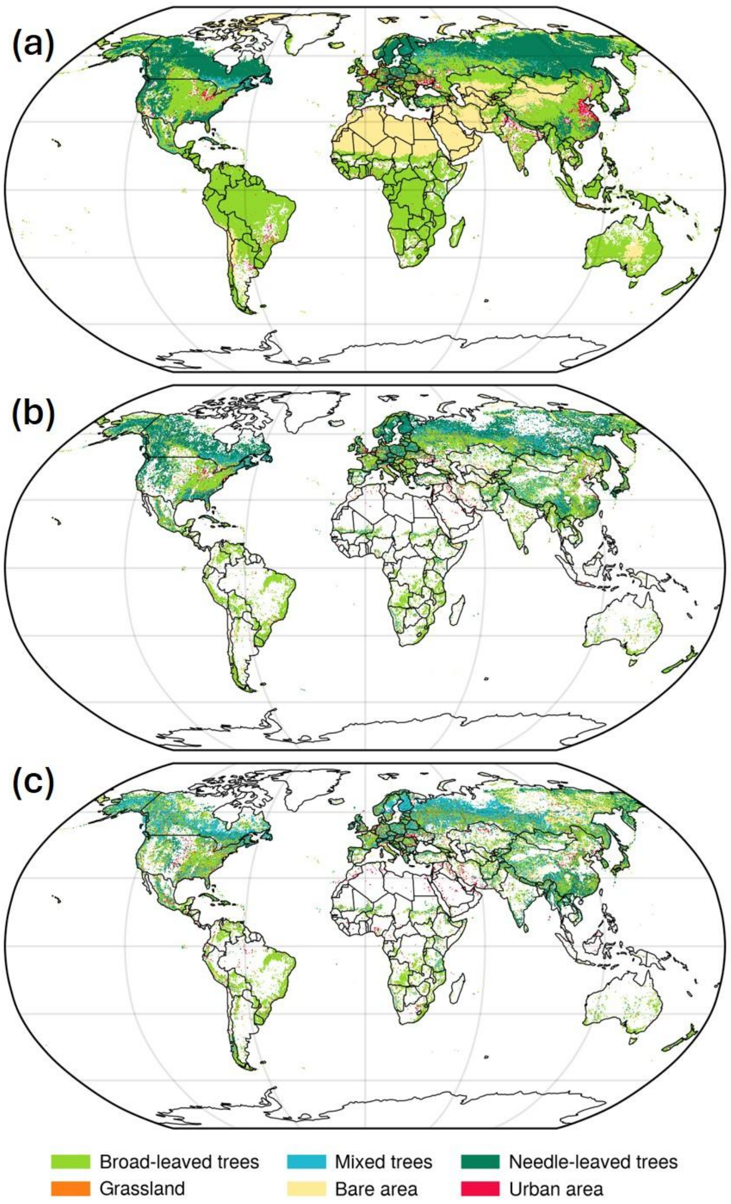

Figure s1. (a) Aggregated pure land covers from the CCI map. (b) Dominant land covers for mixed-land-cover pixels. (c) Second dominant land covers for mixed-land-cover pixels. All of them focus solely on the region between 85°N and 55°S.

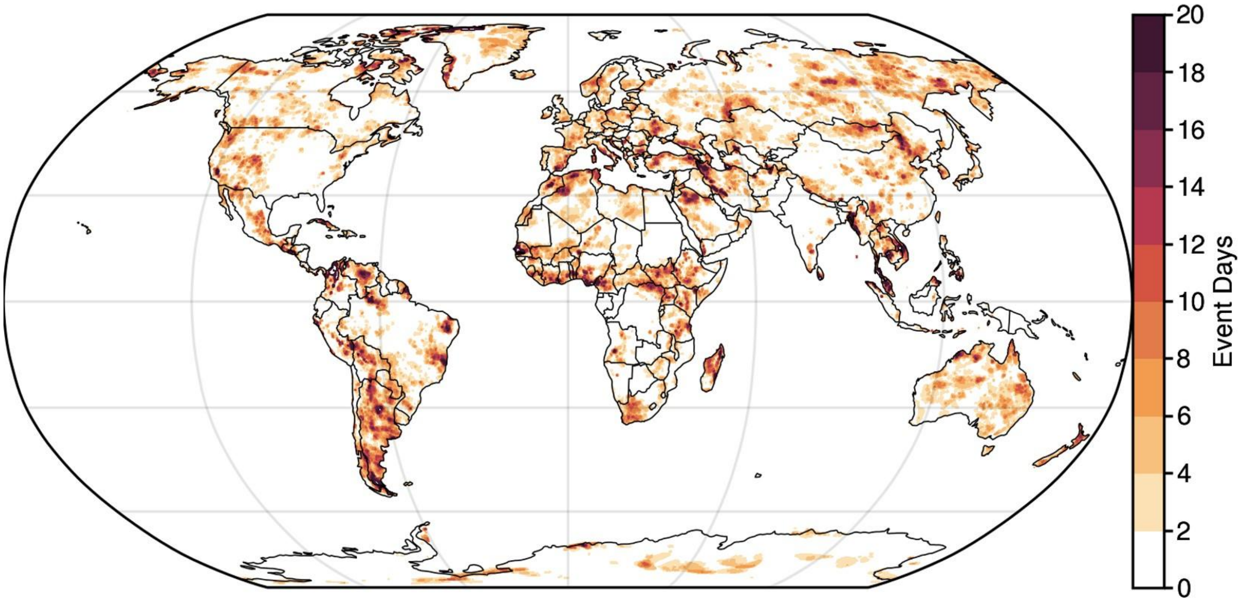

Figure s2. Event days map showing the selected 114 large CHD events from the Dheed dataset.
